# Spatial Structure and Distribution of Small Pelagic Fish in the Northwestern Mediterranean Sea

**DOI:** 10.1371/journal.pone.0111211

**Published:** 2014-11-06

**Authors:** Claire Saraux, Jean-Marc Fromentin, Jean-Louis Bigot, Jean-Hervé Bourdeix, Marie Morfin, David Roos, Elisabeth Van Beveren, Nicolas Bez

**Affiliations:** 1 IFREMER (Institut Français de Recherche pour l'Exploitation de la MER), Research Unit EME (UMR 212), Sète, France; 2 IRD (Institut de Recherche pour le Développement), Research Unit EME (UMR 212), Sète, France; Aristotle University of Thessaloniki, Greece

## Abstract

Understanding the ecological and anthropogenic drivers of population dynamics requires detailed studies on habitat selection and spatial distribution. Although small pelagic fish aggregate in large shoals and usually exhibit important spatial structure, their dynamics in time and space remain unpredictable and challenging. In the Gulf of Lions (north-western Mediterranean), sardine and anchovy biomasses have declined over the past 5 years causing an important fishery crisis while sprat abundance rose. Applying geostatistical tools on scientific acoustic surveys conducted in the Gulf of Lions, we investigated anchovy, sardine and sprat spatial distributions and structures over 10 years. Our results show that sardines and sprats were more coastal than anchovies. The spatial structure of the three species was fairly stable over time according to variogram outputs, while year-to-year variations in kriged maps highlighted substantial changes in their location. Support for the McCall's basin hypothesis (covariation of both population density and presence area with biomass) was found only in sprats, the most variable of the three species. An innovative method to investigate species collocation at different scales revealed that globally the three species strongly overlap. Although species often co-occurred in terms of presence/absence, their biomass density differed at local scale, suggesting potential interspecific avoidance or different sensitivity to local environmental characteristics. Persistent favourable areas were finally detected, but their environmental characteristics remain to be determined.

## Introduction

Because animal spatial distribution is often strongly associated with population dynamics, spatial indices may provide valuable tools for assessing the status of these populations, and in particular the status of endangered or exploited species [1]–[4]. At the population scale, spatial distribution can be seen, in the absence of substantial anthropogenic impacts, as the emergent property of habitat selection. In addition to social motivation (presence *vs.* absence of conspecifics; [5]), spatio-temporal aggregation patterns in animal populations may be explained by individuals sharing similar needs and dealing with similar biotic and abiotic pressures, such as prey abundance *vs*. predation risk [6], propitious *vs*. detrimental environmental conditions [7]. Nonetheless, as population density increases, so does intra-specific competition [8], and individuals are expected to spread towards less suitable habitats when a certain threshold is reached (‘Basin hypothesis’; [9]). Both population density and its occupation area should then vary with its abundance [10]–[12]. If their density-dependent and density-independent drivers are difficult to disentangle (but see [13]), spatial distributions in themselves offer valuable information for both ecological understanding and management.

Indeed, besides an obvious fundamental interest in terms of population dynamics and marine ecosystem functioning [14], information on fish biomass location is also of crucial importance for stock management. For instance, the effective implementation of Marine Protected Areas (MPAs) requires detailed knowledge on species spatio-temporal dynamics, *i.e.* on temporal variability in fish spatial distributions [15]. In addition, knowledge on interspecific interactions, such as the co-occurrence or repulsion of species based on their spatial distributions, may greatly help scientists to understand competition or predator-prey processes but also policy makers to implement ecosystemic rather than single species management measures, as presently done.

In this study, we investigated the spatio-temporal distribution of small pelagic fish in the Gulf of Lions using a unique dataset of 10 years of acoustic surveys. Because small pelagic fish are key species of the pelagic ecosystem due to their central place in the food web, transferring energy from the lowest trophic levels (plankton) towards top-predators [16], and because their life-history traits (short lifespan, large fecundity) make them strongly dependent on the abiotic environment [17], changes in their distribution and abundance have been a major source of concern for scientists and managers worldwide ([18]; Global Ocean Ecosystem Dynamics program - GLOBEC;).

Over the past years, important changes in the two main target species (the European sardine, *Sardina pilchardus* and the European anchovy, *Engraulis encrasicolus*) biomass [19], along with a shift in the size-distribution of these species towards smaller individuals [20] have been observed in the Northwestern Mediterranean Sea (Gulf of Lions), resulting in important economic losses for fisheries. Both stocks are now considered to have a low biomass and a low fishing mortality by General Fisheries Commission for the Mediterranean [19]. In parallel, a third small pelagic species, the sprat (*Sprattus sprattus*), which is not commercially exploited in the Western Mediterranean has appeared in the system with a steadily increasing biomass since 2007 [19]. This unexpected situation offered us the opportunity to describe and compare simultaneous spatio-temporal distributions of three species sharing the same trophic level, with similar feeding behaviours [21]–[22], but with completely different trends in biomass, through the analysis of 10 years of scientific acoustic surveys. First, we studied the aggregation patterns and spatial structures of these species separately. Second, we investigated species spatial dynamics by considering spatio-temporal changes in biomass, and defined optimal recurrent areas based on a combination of biomass levels and their variability. Finally, we focused on interspecific relationships by studying the overlap between species at different spatio-temporal scales.

## Methods

### Ethic statement

The study was conducted in the Gulf of Lions (Longitude in [3.05°; 5.20°] and Latitude in [42.44°; 43.44°]), a public sea area. No sampling was operated from private land and field studies did not involve endangered or protected species. All data used in this study came from PELMED acoustic surveys, which comply with the MEDIAS (Mediterranean Acoustic Survey) protocol. The sampling has been performed under repeated international standardized surveys where the research vessel had full permission to sample from all relevant national public authorities (governments). Acoustic data were collected at a distance, which does not require any particular ethic approval. Further data used in this study came from scientific trawls conducted during these same PELMED surveys. Again, no approval by an ethic committee was required as the targeted species are exploited species and trawling methods done according to international standard trawl surveying.

### Data collection and survey design

Every July since 2003, the French Research Institute IFREMER has been carrying out acoustic surveys of the pelagic resources present in the Gulf of Lions, Mediterranean Sea. The summer period of the survey corresponds to contrasted biological periods for our 3 species. Indeed, this is the peak of reproduction for anchovies, while sprats and sardines reproduce in winter [23]. Due to the biological cycle of these species, it is important not to extrapolate our results outside the summer period. Sampling was performed along 9 parallel transects, regularly spaced by 12 nautical miles (nm) (see Figure S1 in File S1). Acoustic data were recorded every 1 nm using multi-frequency echosounders (Simrad EK500 and ER60), while travelling at a constant speed of 8 nm.h^−1^. All 4 frequencies were visualized during sampling to help deciding when to trawl for species identification. However, only energies from the 38 kHz (typical frequency used for fish) channel were used to estimate fish density. Acoustic data analyses, such as bottom correction, were later performed using *Movies*+ [24] and *FishView* IFREMER softwares. Species discrimination and echo-partitioning were performed by the combination of echo trace classification and trawl outputs [25]. Species biomass and abundance were finally estimated from species energy using specific target strength (TS = 20 log(L) - 71.2, where L is the length of the fish for all 3 species, see [26] for more details on acoustic surveys and analyses). Main survey features are summarised in Table 1.

**Table 1:** Main PELMED survey features from 2003 to 2012.

Year	Sampling dates	Nb of nm	Nb of EDSU	Nb of trawls	Anchovy biomass	Sardine biomass	Sprat biomass
**2003**	07/07/03-06/08/03	1274	284	27	27 860	126 120	685
**2004**	05/07/04-04/08/04	1174	285	29	25 953	215 560	786
**2005**	08/07/05-07/08/05	1462	294	33	15 962	264 024	1 955
**2006**	09/07/06-08/08/06	1473	288	39	25 658	102 276	772
**2007**	11/07/07-10/08/07	1500	290	43	13 654	88 297	15
**2008**	19/06/08-30/07/08	2151	273	60	23 395	91 546	5 002
**2009**	24/06/09-29/07/09	2173	284	43	30 424	52 977	7 845
**2010**	24/06/10-29/07/10	2000	276	39	23 514	51 819	15 760
**2011**	27/06/11-31/07/11	1704	282	42	25 906	44 926	26 638
**2012**	27/06/12-31/07/12	1172	279	37	39 061	80 537	70 263

Biomasses are indicated in tons, nm stands for nautical mile.

### Analyses

We defined and used different spatial indicators and geostatistical methods described in details below. An overview of these indicators, including their calculation formula, representative scale and biological meaning are summarised in Table 2. A key concept in statistics in general and in spatial statistics in particular is the support of the information, i.e. the geographical area over which measures are recorded [27]–[28]. In this study, the support size was the size of the Elementary Sampling Distance Unit, that is 1 nm. All statistics derived from these data are thus associated to the 1 nm sampling support and, for all of them, their values would have been different if computed at another support. Even though some metrics are labelled like spatial statistics (e.g. space selectivity index, local index of collocation), they are not sensitive to the location of the points in space (i.e. any exchange between two data does not change the result). They are sensitive to the spatial patterns that exist at scales smaller than the support size and that are integrated in the observations. For some of them (e.g. Empirical Orthogonal Functions), it remains possible to represent them as geographical distributions making the confusion even worth.

**Table 2:** Spatial indicators used in the study.

Indicator name	Formula	Spatial scale	Time scale	Biological meaning
***Presence area***				
**Presence area**	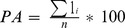	Global	Annual	Area of presence(in %) of the species over the studied area
***Aggregation and spatial structure***				
**Space selectivity index**		Global	Annual	Spatial compactness
**Annual variograms**			Annual	Spatial autocorrelation
**Mean variogram**			Decadal	Mean Spatial autocorrelation
***Spatial distribution***				
**Centre of gravity**	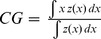	Global	Annual & decadal	Mean location of the species
**Inertia**	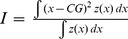	Global	Annual& decadal	Spatial dispersion around CG
**Patches**		Intermediate	Annual	Concentration of biomass
**EOF**		Global	decadal	Spatio-temporal variability
**CV**	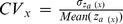	Local	decadal	Temporal variability
***Interspecific relationships/co-occurence***				
**Overlap**	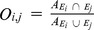	Global	Annual decadal	Overlap between the spatial distribution envelops of two species
**Overlap of patches**	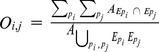	Intermediate	Annual decadal	Proportion of patches where both species present
**Co-occurrence index**	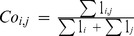	Local	Annual decadal	Level of local co-occurrence between 2 species based on presence/absence
**Local index of collocation**	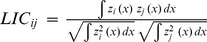	Local	Annual decadal	Level of local cooccurence between species based on densities
**Dominance index**	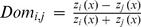	Local	Annual decadal	Relative biomass in each point
***Mapping***				
**Kriging**	Anisotropic neighbourhood	Global	Annual	Distribution maps
***Area classification***				
	Variability map * Average map	Global	Decadal	Identification of recurrent, occasional & unfavourable areas

n: Number of sampling points, z(x): biomass density in x, z_a_ (x): annual biomass density in x.

#### Aggregation patterns and spatial structure

Presence Area (PA) was calculated as the percentage of sampled points at which the species was found, independently of its abundance.

A space selectivity index was defined on the basis of annual concentration curves [29]–[30]. Like Lorenz curves (as used in [31]), concentration curves represent the maximum proportion of biomass as a function of the proportion of samples [30]. For instance, a proportion of biomass of 0.6 for a proportion of sampled area of 0.2 would indicate that 60% of population total biomass was found in only 20% of the total area sampled. For a homogeneously distributed population, each proportion of biomass should be found in the same proportion of total area (i.e. 10% of biomass in 10% of area, etc.), so that the annual concentration curve of a homogeneously distributed population equals the first diagonal. It follows that the more concentrated a population, the further the curve falls from the first diagonal, *i.e.* a higher proportion of biomass is situated in a given proportion of the total area. Consequently, the space selectivity index is defined as twice the area between the concentration curve and the first diagonal [29]–[30], so that the higher the space selectivity index, the more concentrated the spatial distribution of the fish.

Spatial structure was defined as the spatial autocorrelation between values at different locations. In other words, spatial structure considers whether the correlation between biomass in two locations depends on the distance separating them. It was investigated using variograms [27] that calculate the value of the spatial autocorrelation at different lags (distance intervals). As is often the case, the frequency distributions of species biomass were highly skewed with a large proportion of zeros or small values, and few extremely large values contributing importantly to total biomass (coefficients of variation CV were 2.6, 3.8 and 4.2 for anchovies, sprats and sardines, respectively). Besides, the variance in biomass increased proportionally with mean biomass in our data. This led us to log-transform the studied variables (the optimal Box-Cox transformation was **Y = ln (B+c)**, with ***c*** a positive constant added to insure positive values). This constant was set to the smallest biomass observed in our data, *i.e.* 0.05 tons. While distributions of the log-biomass were still skewed, the proportionality effect disappeared. The log-transformed data was used to calculate empirical variograms for all three species for each year of survey. To account for the anisotropy of the sampling (only along parallel transects) and for a potential anisotropic structure due to a bathymetry gradient, bidirectional variograms (along transects and perpendicularly to them) were calculated and modelled by automatic fitting using a least square method [32]. The temporal variability of the spatial structure was assessed on annually standardised data by comparing annual variograms to the mean variogram (average across all years of survey, pairs being retained only if the two sampling locations belong to the same year) and its 95%-confidence interval (adaptation from [12] for bidirectional variograms). The confidence interval was obtained by simulating 1000 random fields according to the mean modelled variogram using the turning-bands method [33] and estimating the 1000 variograms associated with the sampling points extracted from these random fields.

#### Species spatial distributions

To capture the spatial patterns of the 3 populations as simply as possible and to investigate year-to-year variations, we calculated the centres of gravity of their biomass along with their associated inertia [34]–[35]. The centre of gravity represents the mean location of the population, while inertia describes the dispersion of the population around its centre of gravity. Because fish populations are often aggregated in a given area and therefore spatial distributions heterogeneous, we also investigated the presence of spatial patches of high biomass densities, by adapting the recursive algorithm developed by [35]. This was adapted to take into account the anisotropy of our sampling by setting two different threshold distances (one parallel to the transect and another one perpendicular). The sensitivity to the threshold distances was tested using different values. Very small distances did not enable us to identify patches with more than 10% of biomass, while increasing the distance reduced the number of patches to one which comprised the whole presence area. The final distances (i.e. 6 nm in the direction of the transect and 24 perpendicular to it) were set in the interval where the resulting number of patches was stable and were kept constant both across species and years. The number of patches, centres of gravity and inertias of all patches were then used to study year-to-year and interspecific variations.

Kriging maps were produced for each species on a 1 nm*1 nm-grid over the study area, using the modelled mean or annual variograms depending on the results of interannual variability tests. In order to account for anisotropic sampling, we used an anisotropic search of neighbours when conducting kriging, *i.e.* the distance at which neighbours were looked for was smaller in the transect direction than perpendicular to it to make sure that each point comprised neighbours in two different transects.

Two different methods were used to quantify the temporal stability/variability of spatial distributions at 2 different scales (Table 2). First, to assess the stability/variability of the entire distribution, we calculated the Empirical Orthogonal Functions (EOF) on the raw acoustic data for each species. EOF analysis is a decomposition of a spatio-temporal dataset in terms of orthogonal basis functions. It is thus very similar to a Principal Component Analysis, except that it is applied to spatio-temporal data, using time as a descriptor and space (i.e. sampling locations) as objects [36]. EOF was performed on standardized data to compare annual spatial distributions independently of annual total levels of biomass. The first axis (eigen vector) of the EOF is the linear combination of years which maximizes the percentage of the interannual variance of spatial distributions. When all yearly contributions to this first axis share the same sign, the greater the percentage of variance explained by the first EOF, the more persistent the spatial distributions [37]; [12]. In order to have a local estimation of temporal variability/stability and see whether some areas were more stable than others, we also calculated the Coefficient of Variation (CV) of the 10 annual values in each sampling point. As biomass varied substantially between years, we used relative biomass (i.e absolute biomass divided by the total annual biomass) instead of absolute biomass, so that the observed CV corresponded to a change in geographical location rather than a change in overall biomass.

Finally, we defined recurrent, occasional and unfavourable areas by combining average and variability maps [38]. To produce average and variability maps, we calculated the mean and the standard deviation of the 10 annual kriged maps node by node (1 nm*1 nm). Then, each 1 nm*1 nm pixel was assigned one of the 3 categories (recurrent, occasional and unfavourable) depending on its value in the average and variability maps. Pixels with both low mean and variability (i.e. inferior to the median) were considered unfavourable; those with high mean and low variability recurrent, while pixels with high variability were defined as occasional.

#### Interspecific indices: collocation index and overlap between species

We quantified the collocation at three different spatial scales in a coherent manner: from a global scale comparing the distribution over the whole study area, to a local scale comparing species at each sampling point, passing by an intermediate scale comparing previously defined patches.

Global overlap was defined based on centres of gravity and associated inertia ellipses (see above), as follows: 
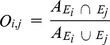
where 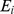 and 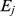 are the inertia ellipses of species i and j respectively and A is the area. The index varies from 0 when the two ellipses are totally separated to 1 when the two ellipses are identical.

At the intermediate scale, we used the same index but calculated from the inertia ellipses associated with the patches of the monospecific distributions: 
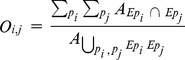
where 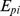 and 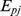 are the ellipses associated with the patches of species i and j respectively.

To characterize the co-occurence of two species at the local scale, we used two different indices: (*i*) an index of co-occurrence defined as a proportion of sampling points where both species co-occur (taking into account only the sampling points where at least one of the two species is present), and (*ii*) a local index of collocation (LIC; [39]) defined as: 
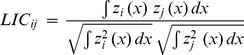
where z_i_ (x) and z_j_ (x) are the biomass density of the species i and j in the location x. Both indices vary between 0 (no location where the 2 species are found simultaneously) and 1 (the two species are situated in exactly the same locations). Yet, they differ in the fact that the former is based only on presence/absence while the second is based on biomass density.

To evaluate the deviation of these indices from random expectation, we used a randomization test by non-parametric bootstrap of 1000 values. For species of strong local overlap (i.e. anchovies and sardines), we also calculated a dominance index based on the relative difference in biomass of the two species in each point (Table 2). This index varies between -1, where only the species j is present and 1 where only the presence i is present, 0 corresponding to a situation where species i and j are equally abundant [40]. This index enabled us to better represent which species dominate where.

#### Softwares

Statistics were conducted using R v. 2.15.0 [41]. Geostatistical analyses were performed with the package RGeoS [42]. Spatial data are given in WGS84 coordinate system. Analyses were conducted on both raw and kriged data. As both methods yielded very similar results, we only presented results on raw data for clarity purposes. Kriging was produced only for mapping purposes.

## Results

### Aggregation patterns and spatial structure

**Figure 1:**
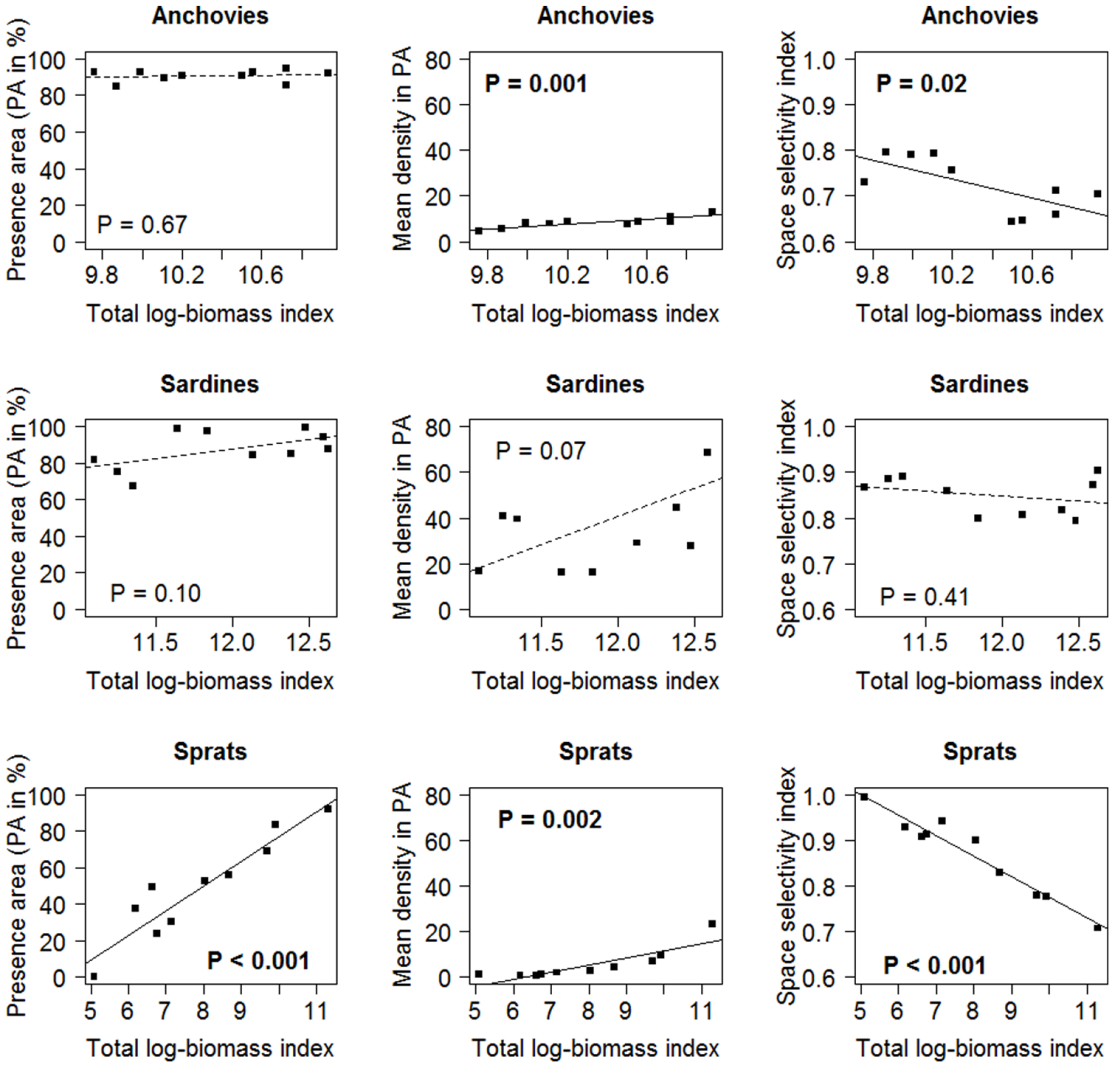
Presence areas, biomass densities and space selectivity indices relatively to total log-biomass indices in anchovies, sardines and sprats. Lines represent the linear regressions. Significant linear regressions are represented by plain lines and Pvalues are indicated in bold. For non-significant relationships, the trend is shown by a dotted line.

Species were absent from several sampled locations, regardless of whether anchovies (absent from 9.3% of sampled locations), sardines (12.9%) or sprats (51.0%) were considered. However, these absences were not spatially consistent across years. When pooling all data, anchovies and sardines could be observed at least once in all sampled locations; sprats in contrast were never found after the 200 m isobath (Fig. S1 in File S1). During each of the 10 study-years, sardines and anchovies occupied most of the Gulf of Lions, and presence area only varied slightly for these two species (PA: 85.0 to 94.7% and 67.2 to 99.6% for anchovies and sardines, respectively). Variability was far greater for sprats, which were almost completely absent in 2007 (PA = 0.3%), but covered most of the Gulf of Lions in 2012 (PA = 91.8%). Presence area was positively correlated with total log-biomass index for sprats (LM: R^2^_adj_ = 0.87, p<0.001), but not for anchovies (p = 0.68) or sardines (p = 0.10) (Figure 1), *i.e.* the area occupied by the population expanded with total logbiomass in sprats but not in the other two species. Log-biomass and presence area were far less variable for sardines and anchovies than for sprats (CVs being at least 4 to 7 fold smaller for PA and biomass, respectively). The mean density in presence area also increased with log-biomass for anchovies (R^2^_adj_ = 0.71, P = 0.001) and sprats (R^2^_adj_ = 0.69, P = 0.002). The same trend was observed in sardines, but the probability was lower (R^2^_adj_ = 0.27, P = 0.07).

The degree of aggregation appeared to be different in anchovies compared to sprats or sardines (Figure 1; Figure S2 in File S1). Anchovies were, on average, less concentrated than the two other species. This was confirmed by the space selectivity index, which was lower in anchovies (0.722) than in sardines and sprats (0.850 and 0.867 respectively). Over the 10 study-years, yearly space selectivity indices decreased significantly with increasing log-biomass in anchovies (LM: R^2^_adj_ = 0.43, P = 0.02,) and in sprats (R^2^_adj_ = 0.94, P<0.001), but not in sardines (P = 0.41, Figure 1).

**Figure 2:**
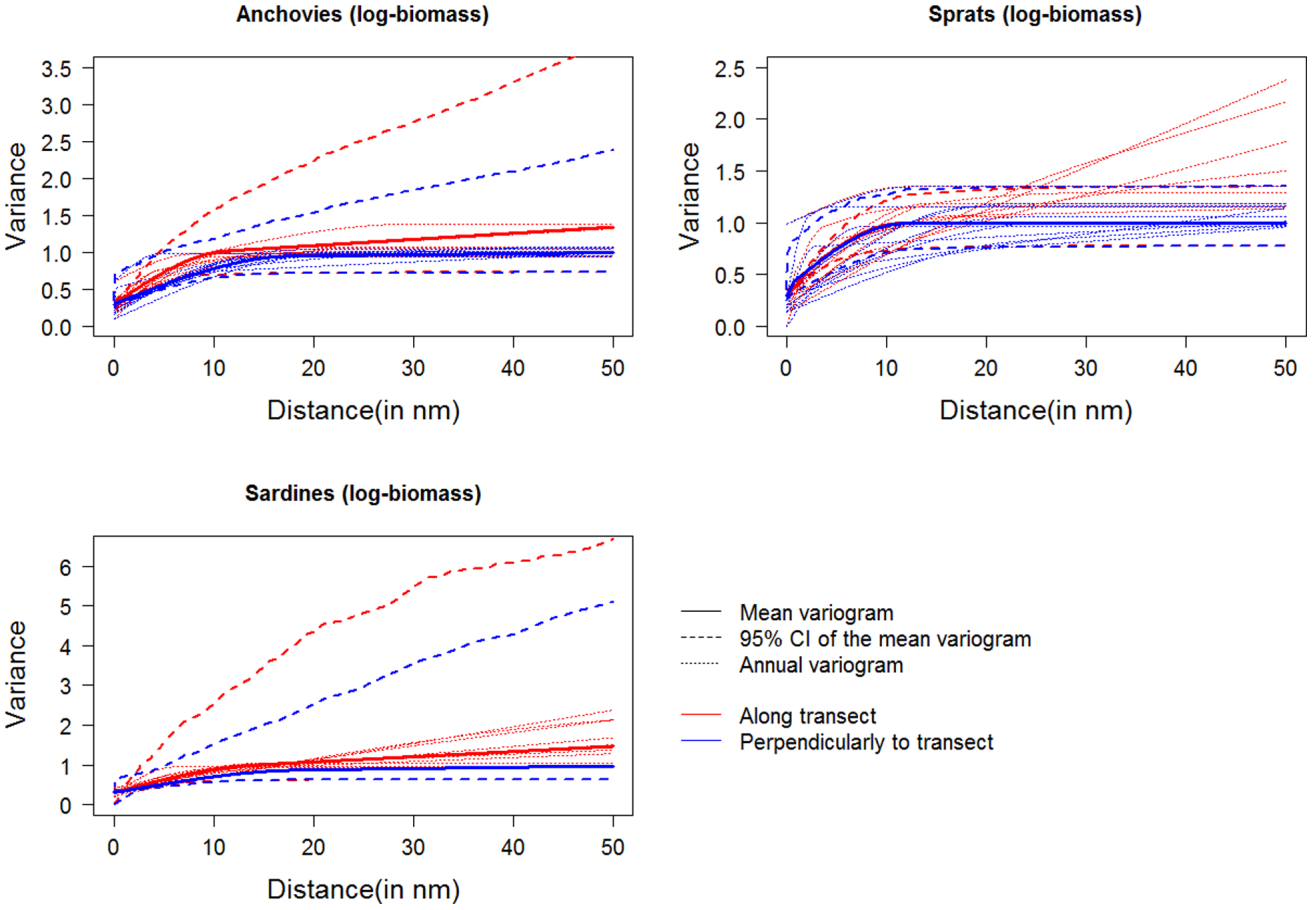
Annual (black) and mean (red) modelled variograms of anchovies, sardines and sprats. The red dotted lines correspond to the 95% confidence interval of the mean variogram deduced from 500 simulations.

Mean variograms of log biomass exhibited spatial structure for the three species in both directions (i.e, increasing variance when the distance between sampling points increased before levelling off; Figure 2), meaning that the spatial distribution was not random and that two close points had a higher probability to have similar values than distant points. While sardine and anchovy variograms shared similar main characteristics, sprat mean variogram differed slightly. In particular, it differed in the range at which variance stabilized (Figure 2). Variance stabilized rapidly in sprats (range = 4 and 12 nm respectively for the first and second spherical component), while the first structure appeared around 15 nm for sardines and anchovies and the second was not apparent at the scale of our study, *i.e.* variance did not stabilize completely within the 50 nm limit that we fixed. Further, the mean structure seemed similar between the two perpendicular directions in sprats, while it differed slightly in anchovies and sardines, though the mean variogram of each direction was mostly included in the confidence interval around the mean variogram of the other direction.

Annual variograms were computed for each of the three species (Figure 2). All exhibited clear spatial structure and were included in the 95% confidence interval around the mean variogram for anchovies and sardines (see Figure 2). This revealed the absence of a year effect on the spatial structure of these 2 species. In sprats however, some variograms (especially along transect) were not included in the confidence interval, suggesting interannual variability in spatial structure.

### Species spatial distribution

#### Log-biomass distributions

The annual spatial distributions exhibited similarities between species in some years (*e.g.* in 2011 a large area in the middle of the shelf was unoccupied both for anchovies and sardines), but not in others (Figures S3, S4, S5 in File S1). Some consistent differences between species also appeared. For instance, anchovies occupied the centre of the continental shelf, while sardines were more coastal. For the three species, kriged annual distributions also revealed the important interannual variability of these populations both in terms of biomass levels and their spatial repartitions.

Centres of gravity of spatial distributions did not vary much according to species or year and were situated close to the geometrical centre of gravity of the sampled area (8.2±0.8 nm). Interestingly, the associated inertia was high for anchovies and sardines (inertia = 1152±70 nm^2^), meaning that the mean distance between a presence point and the centre of gravity of its population was 34 nm. This distance was remarkably close to that between a sampling point and the geometrical centre of gravity of the total area (37 nm). As a consequence, the ellipse associated with this inertia covered most of the sampled area for these two species (Figure S6 in File S1), *i.e.* the populations were dispersed in the entire Gulf of Lions. In sprats, while inertia was quite low in the first 6 years (447±101 nm^2^; Figure S6 in File S1) due to their low presence, it reached values almost as high as the ones in sardines and anchovies, in the last 4 years (991±75 nm^2^).

**Figure 3:**
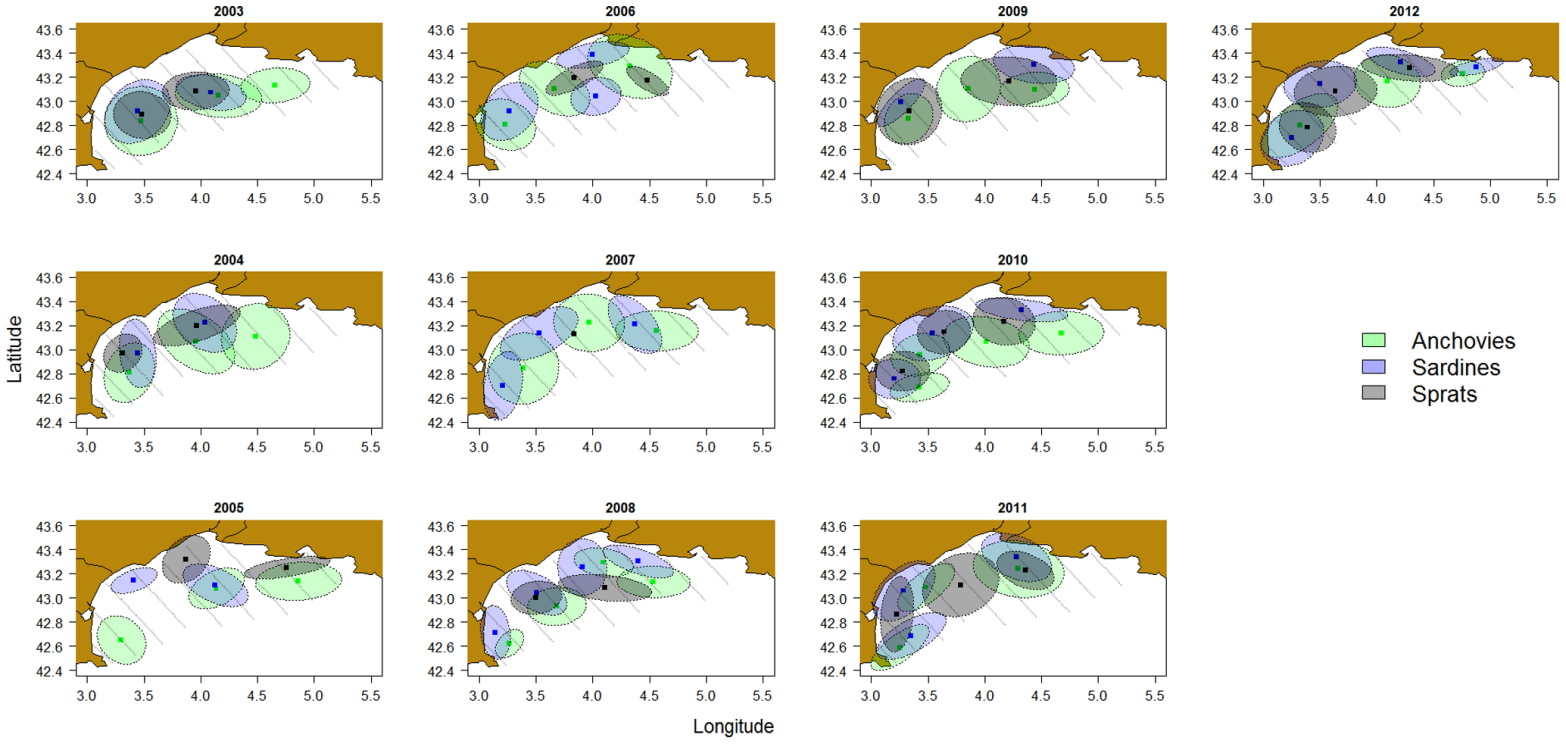
Annual maps of centres of gravity and inertia of patches for anchovies (in green), sardines (in blue) and sprats (in black).

The number of patches present per year and per species varied between 1 and 4 (Figure 3). As inferred by the square root of the inertia associated with the patch, the mean distance between a presence point in a patch and its centre of gravity ranged from 6 nm to 17 nm (excluding 2007 in sprats for which the species had only been observed in 1 location). The centres of gravity of anchovy patches were slightly more offshore than those of sardines (14.9±0.8 vs. 11.1±1.0 nm, Wilcoxon test: W = 662, P = 0.001), confirming the tendency of sardines to be more coastal than anchovies.

#### Instability of the spatial distribution

EOF of the 10 annual maps of each species confirmed the visual inspection highlighting high temporal variations in the spatial distributions. The first component of the EOF explained no more than 20% of the variance (14%, 20% and 17% for anchovies, sardines and sprats, respectively). Further, no trend could be detected from the contributions of each year to the first 2 axes of the EOF. The sign of the correlations between annual maps and the two first axes varied depending on the year for the three species (Figure S7 in File S1).

Coefficients of variation (calculated in each sampling point) were higher in sprats than in sardines and anchovies. Most coefficients of variation were higher than 1 (100%, 92.3% and 81.6% of CV values were higher than 1 in sprats, sardines and anchovies, respectively), meaning that the distributions were overdispersed and again suggesting high temporal variability of the spatial distributions for all three species.

#### Recurrent, occasional and unfavourable areas

**Figure 4:**
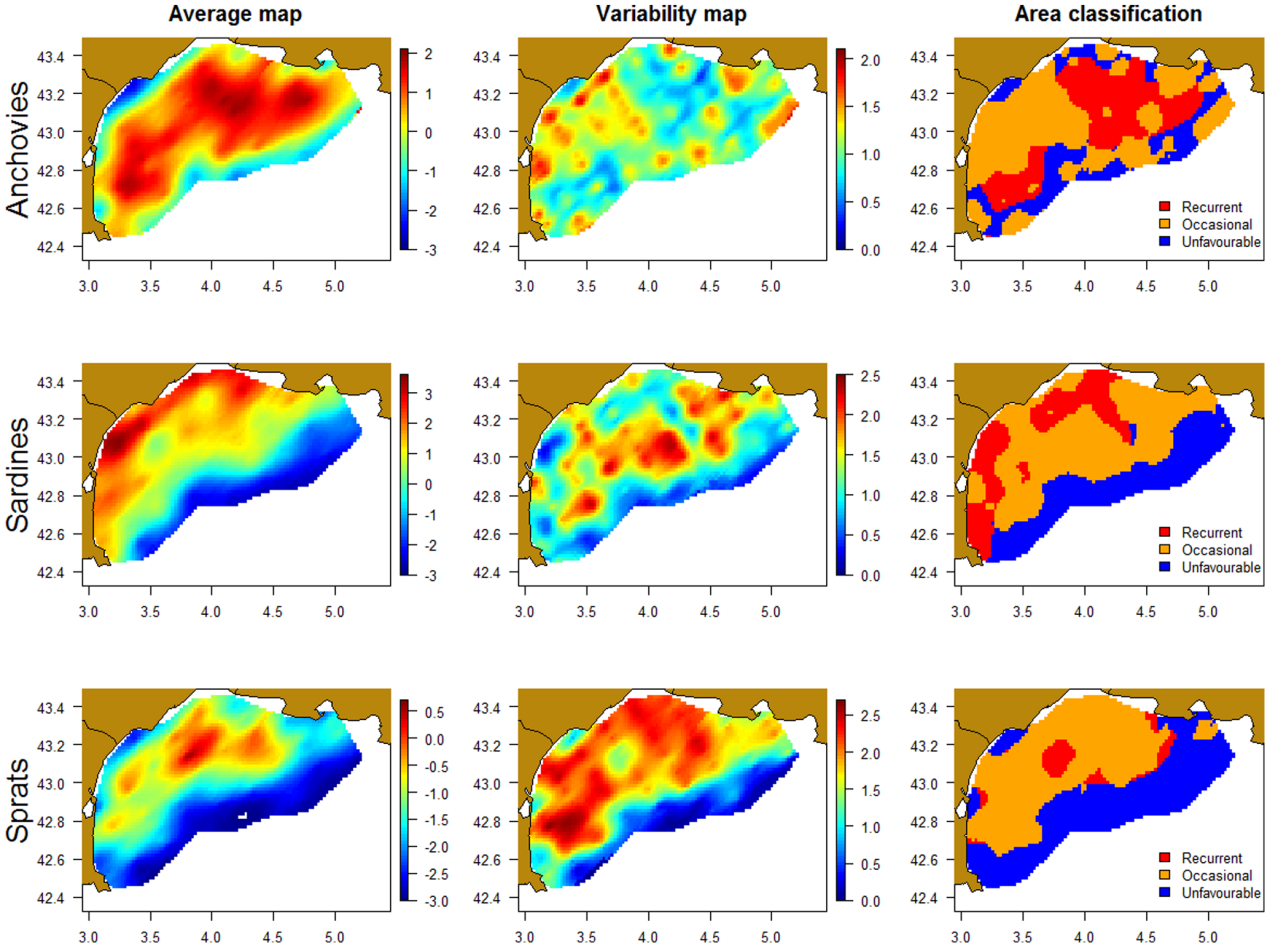
Average and variability maps and area classification for anchovies, sardines and sprats.

Average maps confirmed the tendency for sardines to be quite coastal, while anchovies occupied most of the shelf (Figure 4). Sprats seemed in an intermediate position where high biomass areas were situated in the centre of the Gulf of Lions, neither too coastal nor too offshore, but, as noted above, sprat biomass was also more variable. The combination of average and variability maps enabled us to detect a recurrent (persistent) area for anchovies in the centre of the Gulf, slightly West of the Rhone estuary, and, for sardines, in western coastal areas. Recurrent areas were minimal for sprats as the period encompassed some years with a quasi-absence of this species. Finally, deep waters represented unfavourable areas both for sardines and sprats.

### Interspecific indices: collocation index and overlap between species

Regardless of the scale at which we investigated it, collocation indices were higher between sardines and anchovies than between any of these 2 species and sprats (Table S1 in File S1), meaning that sardines and anchovies co-occurred more often than they did with sprats. Additionally, collocation indices were higher, on average, at the global scale than at the intermediate scale (*e.g.* between anchovies and sardines 0.53±0.04 *vs.* 0.30±0.04 for the global and intermediate scales, respectively), suggesting that species globally lived in the same areas, but were not always found together in each given sampling location. Collocation indices, whether global, local or intermediate, varied substantially from year-to-year (Table S1 in File S1). Collocation indices at the three different scales were not correlated with each other, except for the global and intermediate indices between sprats and anchovies. For instance in 2004, the index of collocation between sprats and sardines was fairly high at the global scale (2^nd^ highest observed), while the associated LIC was low and not significantly different from random expectation. The two local collocation indices were not correlated for anchovy and sardine association or anchovy and sprat association (ρ = −0.61, P = 0.07; ρ = 0.44, P = 0.20 respectively), suggesting that the co-occurrence of species (i.e. species found in the same location), did not necessarily concur with a co-occurrence of their hotspots or peaks of biomass. They were however slightly correlated when looking at the association between sardines and sprats (ρ = 0.72, P = 0.02).

**Figure 5:**
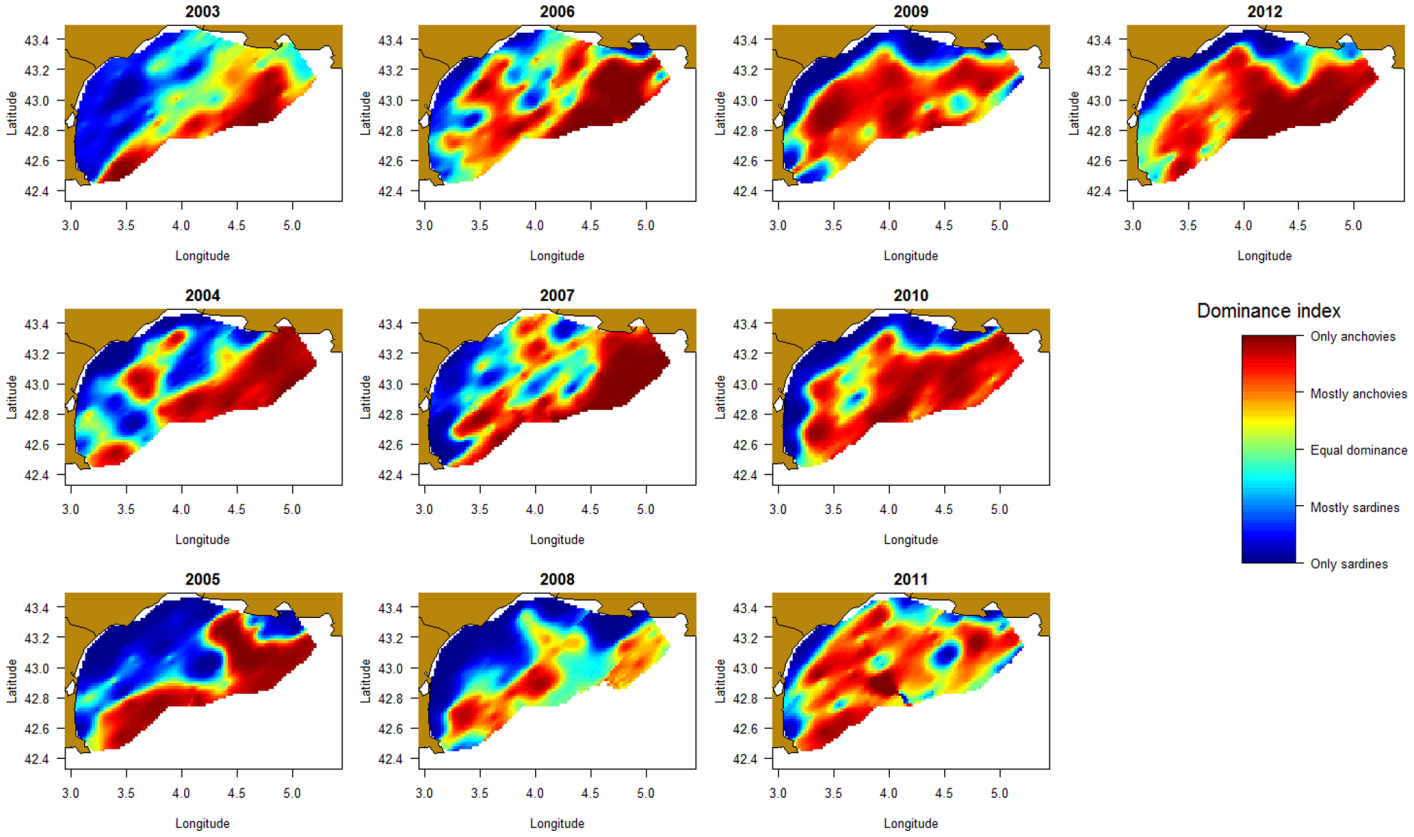
Annual maps of relative biomass between anchovies and sardines.

Finally, although the co-occurrence between sardines and anchovies was high (Table S1 in File S1), in most sampled points one of the two species clearly dominated the other (Figure 5). The dominance index increased with depth, *i.e.* anchovies dominated sardines in deeper offshore waters, while sardines were dominant in shallow waters close to the coast (see also statistics in Figure S8 in File S1). Besides, the dominance index also increased with longitude, anchovies dominating towards the East (see statistics in Figure S8 in File S1).

## Discussion

The strong increase in sprat biomass (from almost 0 to 70,000 tons from 2007 to 2012) that has paralleled a strong decline in anchovy and sardine biomass over the past few years [19], highlighted the urgent need to better understand the cross and mutual dynamics of those three species. This study documents the spatio-temporal patterns observed in these three species in the Gulf of Lions. However, it should be noted that surveys were conducted only in summer, corresponding to peak reproduction of anchovies but resting period for sardines and sprats. The results presented here thus correspond to different biological phases for the three species and should not be extrapolated to other non-monitored seasons.

Our main objective was to investigate the temporal variability of biomass and spatial distribution in the three species. Biomass estimates obtained by direct acoustic stock assessment substantially varied during the 10 years of survey. However, sprat was the only species in which a clear trend in biomass could be identified over the study period. For anchovies and sardines, biomass variability corresponded, respectively, either to fluctuations around a low value or a succession of an increasing and decreasing trends. The increase in sprat biomass resulted in both an increase of fish local densities and an expansion of fish towards new areas, in agreement with the McCall basin hypothesis [9]. On the contrary, the Gulf of Lions being a favourable area for small pelagics [43], sardine and anchovy presence area was large and stable over the entire study period and fluctuations in biomass only resulted in fluctuations of local densities. Changes in local densities could be a buffer for fluctuating biomasses of already well implanted species. However, if a species continuously increase or decrease its biomass, this might well not be enough anymore. For instance, sprats which were almost absent from the area at the start of the study had to expand their presence area to cope with their increasing biomass. Inversely, a declining population would see local areas disappear from its presence area as these species are gregarious and have to stay in shoals. Our results may thus support the hypothesis that interannual variability may not be enough to observe contraction/expansion, and that a directional trend would be needed to investigate the link between abundance and distribution [44]–[45]. Still, using space selectivity indices to investigate aggregation patterns in more details, we found that sprats and anchovies became less selective with increasing biomass, suggesting that they selected less suitable areas rather than increased their local density. This is in contrast with sardines for which the space selectivity index did not vary according to total biomass and densities 5 fold higher could be reached. This could suggest different social constraints/benefits, sardines being able to aggregate in much denser shoals than the other 2 species.

Similarly to what was found in demersal species [12], we highlighted the stability of the spatial structure (i.e. spatial autocorrelation) of small pelagic fish in the Gulf of Lions over time. Only for sprats did some interannual variability appear, but this is probably due to a reduced confidence interval around the mean variogram due to low and distance-independent variance between sampling points in the first half of the study, when sprats were quasi-absent. Spatial structure is defined as the spatial autocorrelation between values at different locations, i.e. in our case whether biomasses taken at 2 close points were more correlated than biomasses of points situated far apart. From an ecological point of view, spatial structure could thus indicate the occurrence of concentration in certain areas due to similar needs (e.g. habitat preferences) or aggregation patterns due to species social constraints (formation of shoals for instance here). Such stability of the spatial structure could result from important aggregative behaviours of small pelagic species, giving them intrinsic aggregation properties, independently from their abiotic environment.

Though necessary, the study of the spatial structure offers no insight on the locations where these aggregations took place or where the population was absent. In a second step, we thus investigated temporal changes in the geographic locations of biomass. In contrast to the stable spatial structure, the geographical distributions of small pelagics were highly variable from year-to-year. Although such variability is clearly visible when comparing the maps, it is more difficult to quantify it. Therefore, we resorted to spatial indicators (CVs and empirical orthogonal functions) for summarizing annual information and exploring possible spatial shifts in population distribution [35]; [46]. These indicators confirmed the visual inspection of the annual maps (high interannual variability) and thus the expected mobility of small pelagic fish, which contrasted to the stable and area-specific spatial distributions of demersal species of the same area [12]. Such result was to be expected, as demersal species are restricted to the bottom and very dependent on substrates or bathymetry. By contrast, small pelagic fish live in the water column, are much more mobile and should be affected mostly by dynamic environmental factors, which change from year to year. Previous studies on small pelagic fish habitats conducted in the Mediterranean [43] have identified the Gulf of Lions as a potential favourable habitat for sardines and anchovies. In this smaller-scale study, we tried to identify whether there could be within Gulf variations in habitat quality for small pelagic fish. From the presence/absence data pooled over the entire study period, we saw that the three species could use the entire Gulf of Lions (except past the 200 m isobath for sprat). Nonetheless, it was clear that spatial distributions were not uniform over the Gulf and that they exhibited more than one patch of high biomass. The location of patches varied across years and between species, suggesting that they reacted to some external variable factors. In the future, linking spatio-temporal distributions to environmental variables at a minute scale while accounting for aggregation structures should help in understanding the environmental drivers affecting the population dynamics of those three pelagic species. Previous studies on environmental drivers of small pelagic fish habitats have already been conducted at a large scale in the Mediterranean, showing the importance of temperature and chlorophyll concentration as forcing factors of the spatial distribution (*e.g.* [43]). However, processes that drive the small pelagic populations may be different at smaller scales (i.e. within the Gulf of Lions). In particular, the relative importance of density-dependent *versus* density-independent (environmental) processes on spatial distributions is likely to be dependent on the scale of the study [47]. A study accounting for both density-dependent and independent variables is thus of necessity to understand the identified areas [13].

Despite the important variability in spatial distributions, we defined unfavourable areas close to the coast for anchovies and on the 150–200 m-deep stratum for sprats and sardines. In contrast, we could assess recurrent areas (high average biomass and low variability) for sardines and anchovies in the Gulf of Lions. For sardines, recurrent areas were situated near the coast and in the Western part of the Gulf of Lions. For anchovies, two recurrent areas were identified further from the coast (isobaths of ∼70 to 100 m). One was situated on the west part while the other one could be associated with the Rhone river and its plume on the east. As the surveys occurred during peak reproduction for anchovies [23], it is likely these areas corresponded to the spawning grounds of anchovies in this region. Interestingly, these 2 areas are very similar to the spawning areas detected from egg surveys in the 60 s [48]. This gives us important insights on their spawning grounds, exhibiting their stability despite several changes in external variables between these 2 periods (e.g. fishing effort and captures, environment). Also, such information is important for the elaboration of spatially explicit management plan (e.g. MPA, etc.). Indeed, it shows that despite the high mobility of small pelagic fish and high interannual variability in peak biomass locations, some areas consistently offer favourable spawning grounds for this species, making the task easier if one wanted to protect them.

Finally, we investigated interspecific relationships between 3 species sharing similar trophic level (i.e. zooplankton and especially copepods constitute the bulk of their diet at that period, though phytoplankton is also consumed in particular by sardines [21]–[22]). Different results were obtained according to the scale at which it was studied. Indeed, the index of overlap that we proposed decreased from the global (the population envelop) to the intermediate scale, indicating that if the three species co-habited in the Gulf of Lions, their hotspots were not always situated in the same area. This suggests that global environmental factors may have driven the three species to inhabit the Gulf of Lions, but that local environmental factors and/or inter-specific competition may have resulted in segregation within the Gulf. This was confirmed by the difference obtained between the two local collocation indices used. The collocation index translating species co-occurrence was surprisingly high (especially for anchovies and sardines), revealing that at a 2-nm sampling scale, species almost always co-occurred. The LIC index (based on density rather than presence/absence) had lower values, suggesting that for most sampled locations a given species predominantly occurred in each location (as also indicated by the dominance index). In particular, sardines clearly dominated the first depth strata (0–50 m) and the western part of the Gulf of Lions, while anchovies were more abundant in deeper waters and towards the central and eastern part of the Gulf. Collocation analyses give us interesting information on potential competition of these three planktivorous species, as their co-occurrence is a potential source of competition if/when food becomes limiting.

In summary, this study exhibited the relatively stable aggregation structure of small pelagic species, probably due to their inherent social structure (shoals, etc.). In contrast, it confirmed that the three species were highly mobile and could inhabit every stratum of the Gulf of Lions, though some preferred and unfavourable habitats could be highlighted for each species. The environmental drivers of both these habitats and the variability in locations of peak biomass remain to be investigated to better understand population dynamics. Finally, differences between species aggregative behaviour and habitat preferences were also highlighted, exhibiting potential competition avoidance.

## Supporting Information

File S1 **Supplementary material. Figure S1.** Presence of anchovies, sardines and sprats in the Gulf of Lions. **Figure S2**. Annual aggregation curves for each species. **Figure S3**. Annual maps of log-biomass for anchovies. **Figure S4**. Annual maps of log-biomass for sardines. **Figure S5**. Annual maps of log-biomass for sprats. **Figure S6**. Centres of gravity and inertia. **Figure S7**. Empirical Orthogonal Function analysis. **Figure S8**. Dominance index depending on depth or longitude strata. **Table S1**. Yearly and global collocation indices at three different time scales.
(DOCX)
