# Supplementary material for: Spatial Structure and Distribution of Small Pelagic Fish in the Northwestern Mediterranean Sea
Source: PLoS One. 2014 Nov 6;9(11):e111211. doi: 10.1371/journal.pone.0111211 (PMC4222880; doi:10.1371/journal.pone.0111211)
Supplement: File S1 — Supplementary material. Figure S1. Presence of anchovies, sardines and sprats in the Gulf of Lions. Figure S2. Annual aggregation curves for each species. Figure S3. Annual maps of log-biomass for anchovies. Figure S4. Annual maps of log-biomass for sardines. Figure S5. Annual maps of log-biomass for sprats. Figure S6. Centres of gravity and inertia. Figure S7. Empirical Orthogonal Function analysis. Figure S8. Dominance index depending on depth or longitude strata. Table S1. Yearly and global collocation indices at three different time scales. (DOCX) [file pone.0111211.s001.docx]

***Supplementary Material***

|  | Global  AS | Global  SSp | Global  ASp | Interm AS | Interm SSp | Interm ASp | LIC  AS | LIC  SSp | LIC  ASp | Co-occ  AS | Co-occ  SSp | Co-occ  ASp |
| --- | --- | --- | --- | --- | --- | --- | --- | --- | --- | --- | --- | --- |
| 2003 | 0.457 | 0.454 | 0.530 | 0.514 | 0.596 | 0.393 | 0.397*** | 0.290** | 0.631*** | 0.893*** | 0.442*** | 0.398 |
| 2004 | 0.433 | 0.643 | 0.335 | 0.287 | 0.363 | 0.221 | 0.350* | 0.066 | 0.436*** | 0.924*** | 0.520*** | 0.530 |
| 2005 | 0.366 | 0.334 | 0.125 | 0.176 | 0.052 | 0.078 | 0.401** | 0.123 | 0.042 | 0.808*** | 0.262 | 0.356 |
| 2006 | 0.758 | 0.258 | 0.252 | 0.281 | 0.028 | 0.143 | 0.634*** | 0.031 | 0.052 | 0.760*** | 0.203 | 0.257 |
| 2007 | 0.512 | 0 | 0 | 0.392 | 0 | 0 | 0.550*** | 0.002 | 0.046 | 0.687*** | 0.005*** | 0.004 |
| 2008 | 0.610 | 0.322 | 0.348 | 0.269 | 0.226 | 0.155 | 0.534*** | 0.066 | 0.110 | 0.897*** | 0.526*** | 0.586 |
| 2009 | 0.543 | 0.641 | 0.682 | 0.099 | 0.259 | 0.483 | 0.135 | 0.484*** | 0.494*** | 0.852 | 0.562*** | 0.610 |
| 2010 | 0.463 | 0.475 | 0.415 | 0.227 | 0.476 | 0.306 | 0.160 | 0.328* | 0.277 | 0.929*** | 0.710*** | 0.709 |
| 2011 | 0.589 | 0.665 | 0.608 | 0.424 | 0.284 | 0.348 | 0.336** | 0.270* | 0.225* | 0.876*** | 0.809*** | 0.918*** |
| 2012 | 0.606 | 0.610 | 0.562 | 0.338 | 0.458 | 0.420 | 0.433*** | 0.409** | 0.414*** | 0.793 | 0.809** | 0.893*** |
| **Mean± SE** | **0.534 ± 0.036** | **0.440 ± 0.068** | **0.385 ± 0.069** | **0.301 ± 0.038** | **0.274 ± 0.064** | **0.255 ± 0.050** | **0.393 ± 0.051** | **0.207 ± 0.054** | **0.273 ± 0.067** | **0.842 ± 0.025** | **0.485 ± 0.084** | **0.526 ± 0.090** |

**Table S1.** A stands for anchovies, S for Sardines and Sp for Sprats. Global and intermediate collocation values correspond to overlap indices based on intersections of inertia ellipses of 1) the whole distribution and 2) the patches. Local values correspond to LIC and Co-occ indices and significance of these values compared to 0 are indicated by stars: * P< 0.05, ** P < 0.01; *** P < 0.001

***
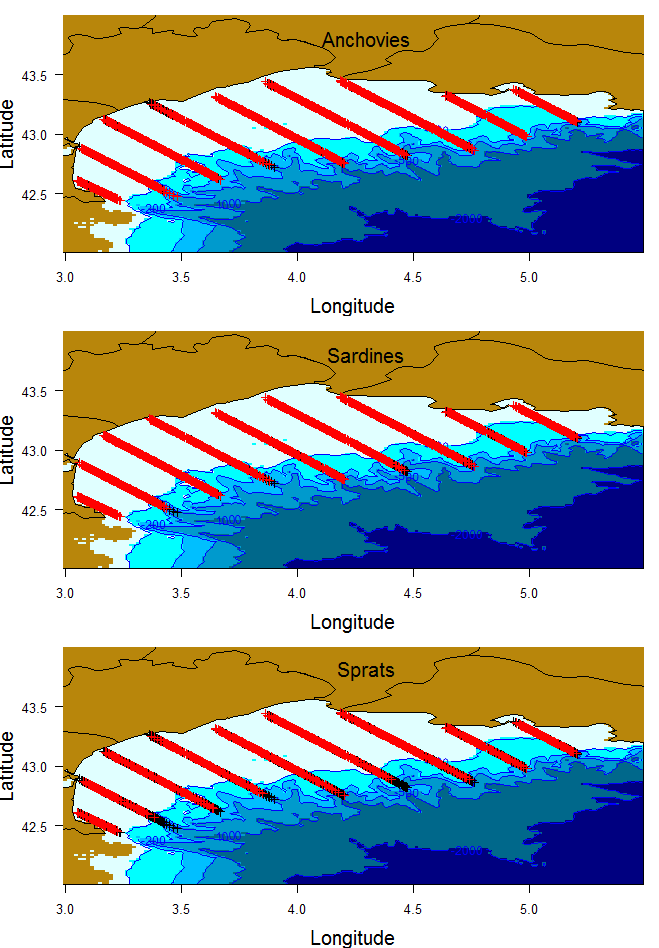
***

**Figure S1.** Presence of anchovies, sardines and sprats in the Gulf of Lions. Red crosses correspond to sampled areas where the species have been detected at least once during the study period, while black ones correspond to sampled areas where the species has never been spotted. Shaded blue represents bathymetry.

***
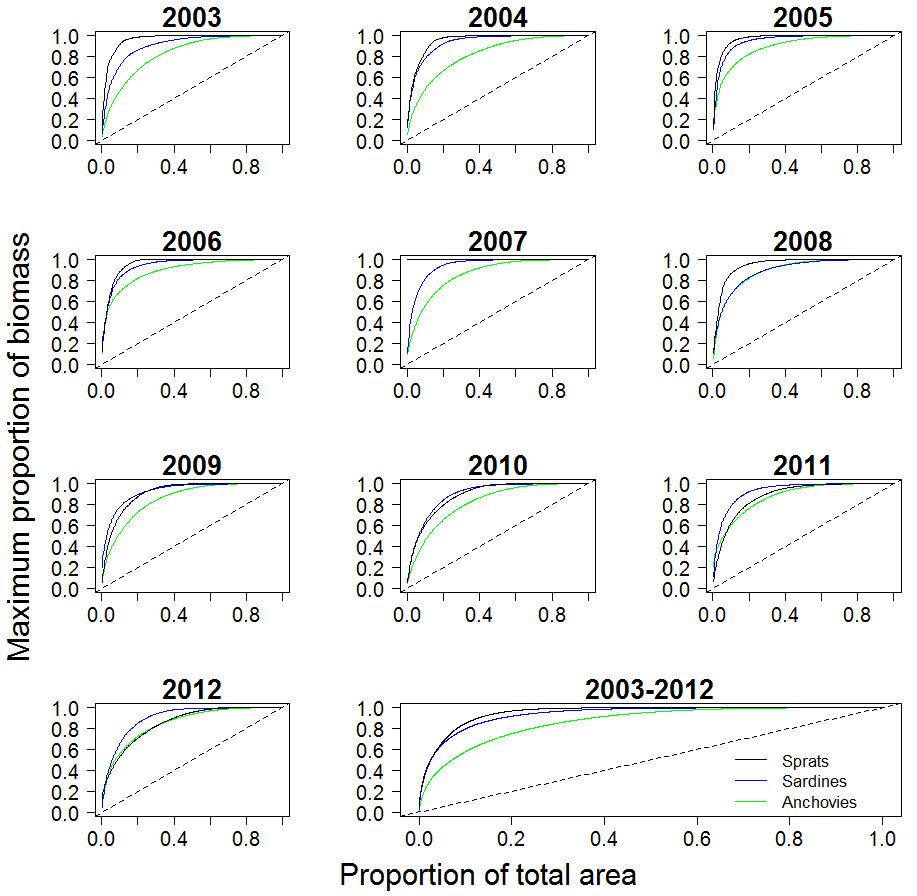
***

***Figure S2.*** Annual aggregation curves for each species (anchovies in green, sardines in blue, and sprats in black)

***
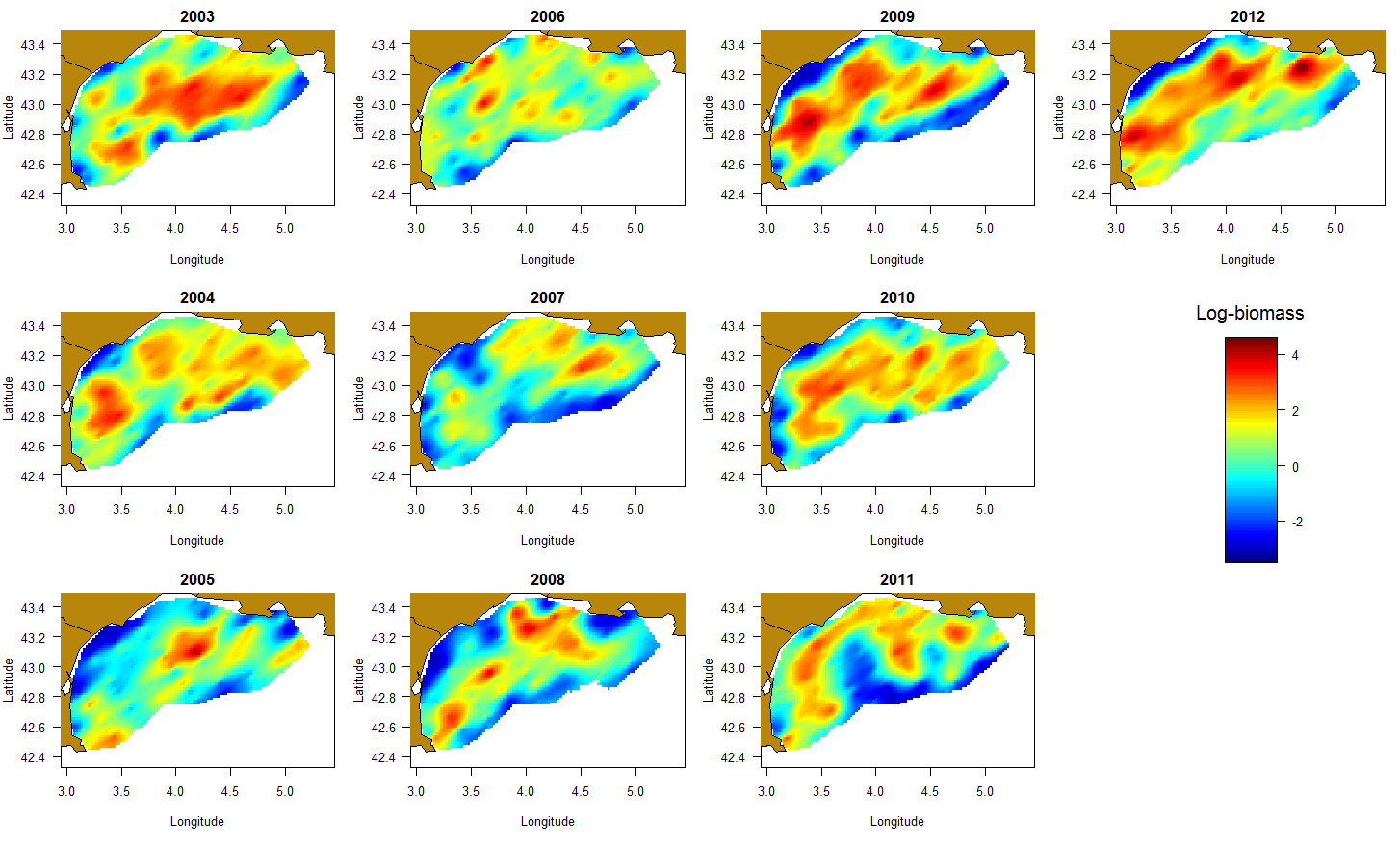
*Figure S3.** Annual maps of log-biomass for anchovies.

**
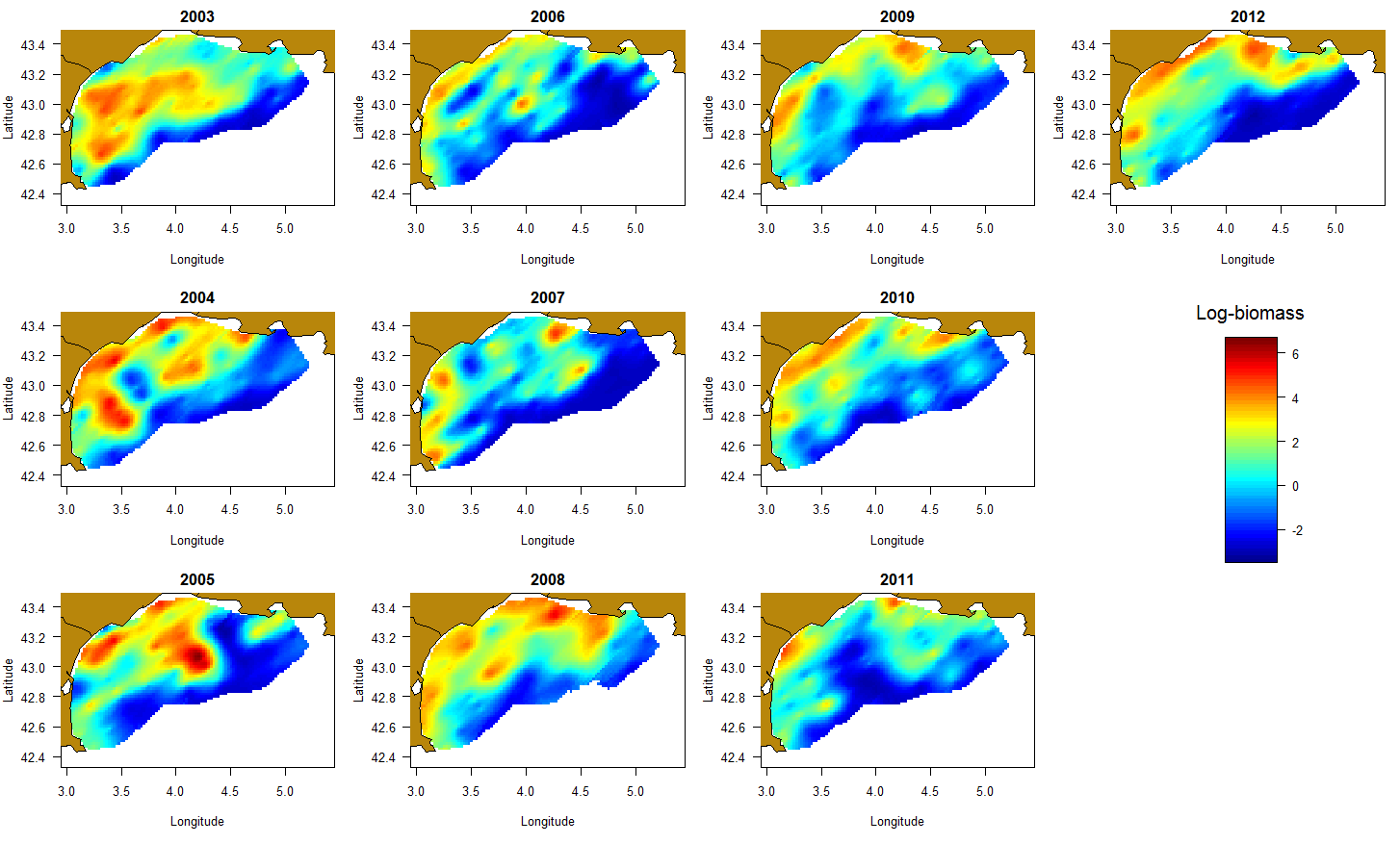
Figure S4.** Annual maps of log-biomass for sardines.

**
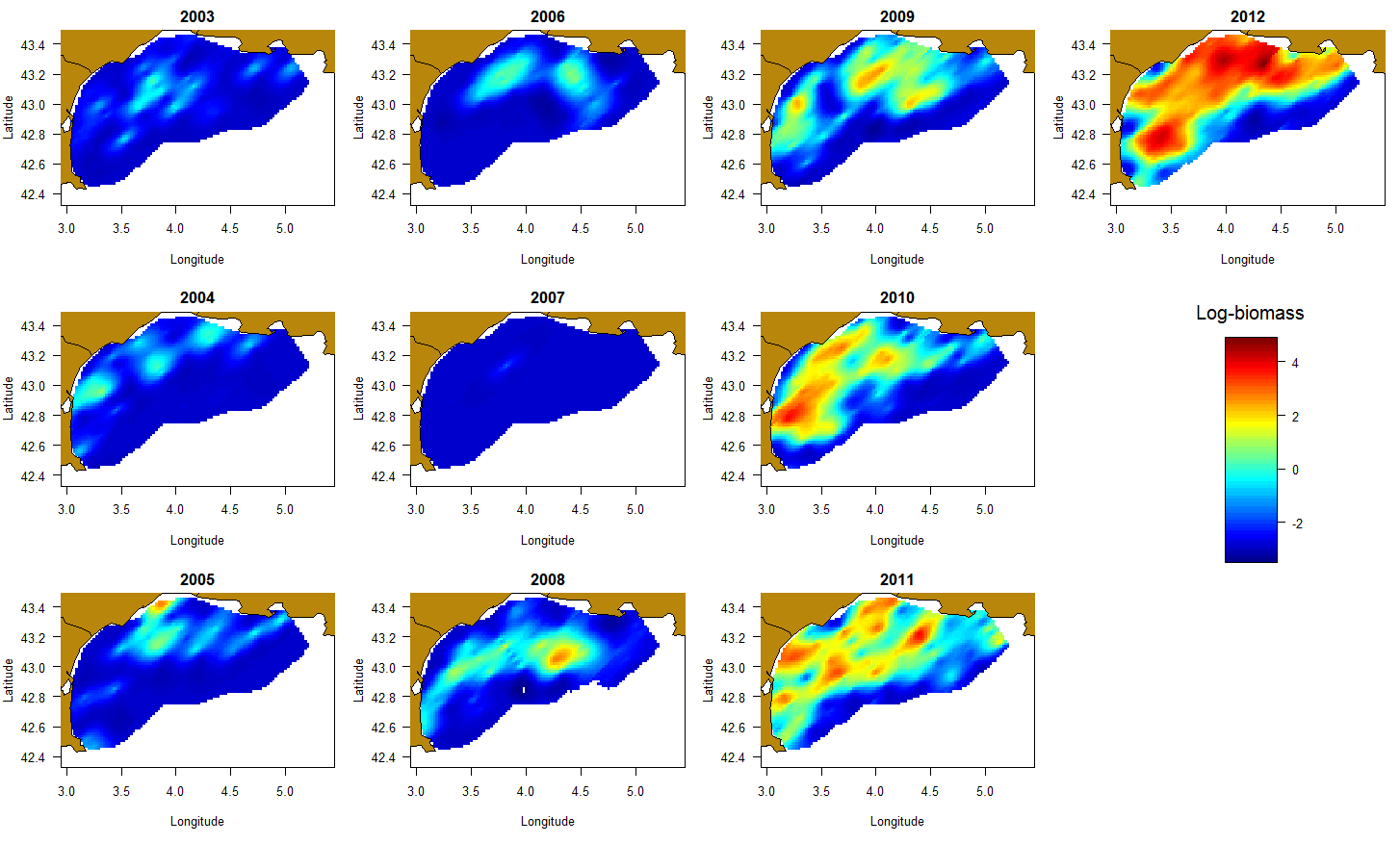
Figure S5.** Annual maps of log-biomass for sprats.


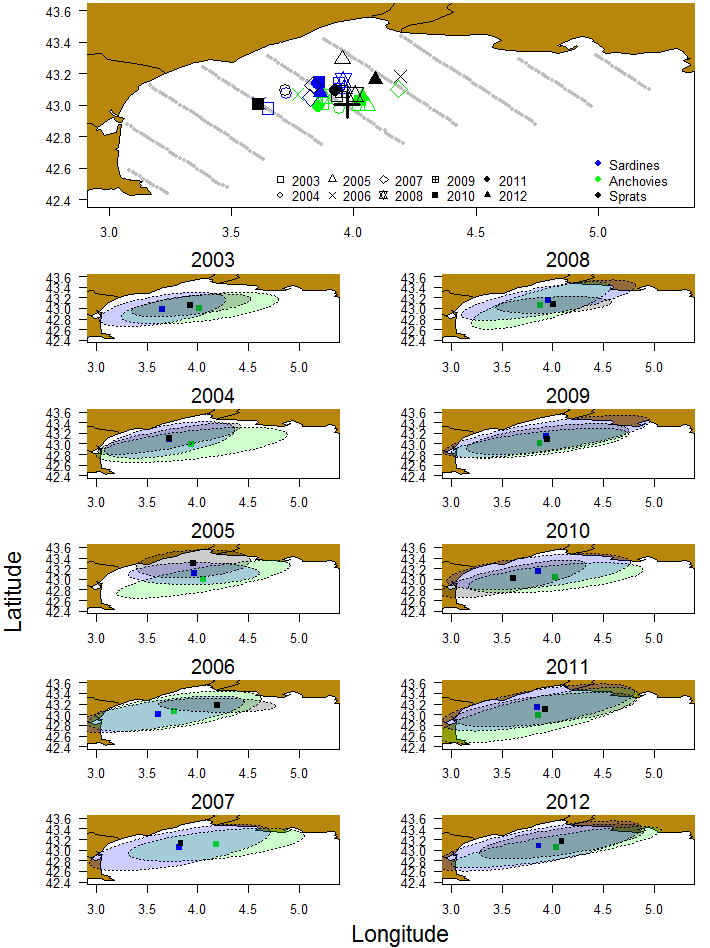


***Figure S6.*** Centres of gravity and inertia. Anchovies are represented in green, sardines in blue, and sprats in black. The first panel shows all annual centres of gravity compared to the centre of the sampling zone (black cross). The other panels show for each year the centres of gravity and associated inertia (ellipses) of all 3 species.

***
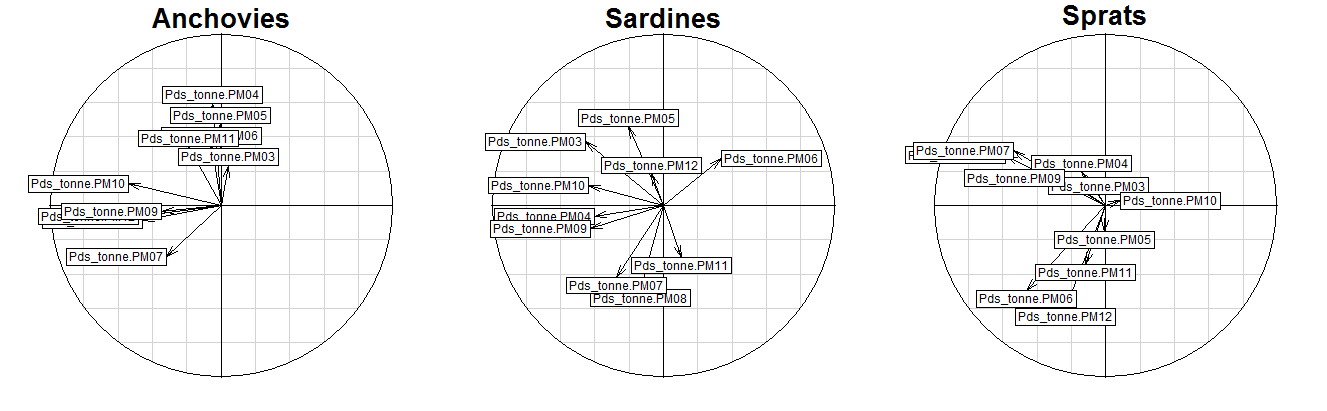
***

***Figure S7*** Empirical Orthogonal Function analysis. Projections on the first two axes of the EOF analysis performed on raw data of log-biomass of each species from 2003 to 2012.

***
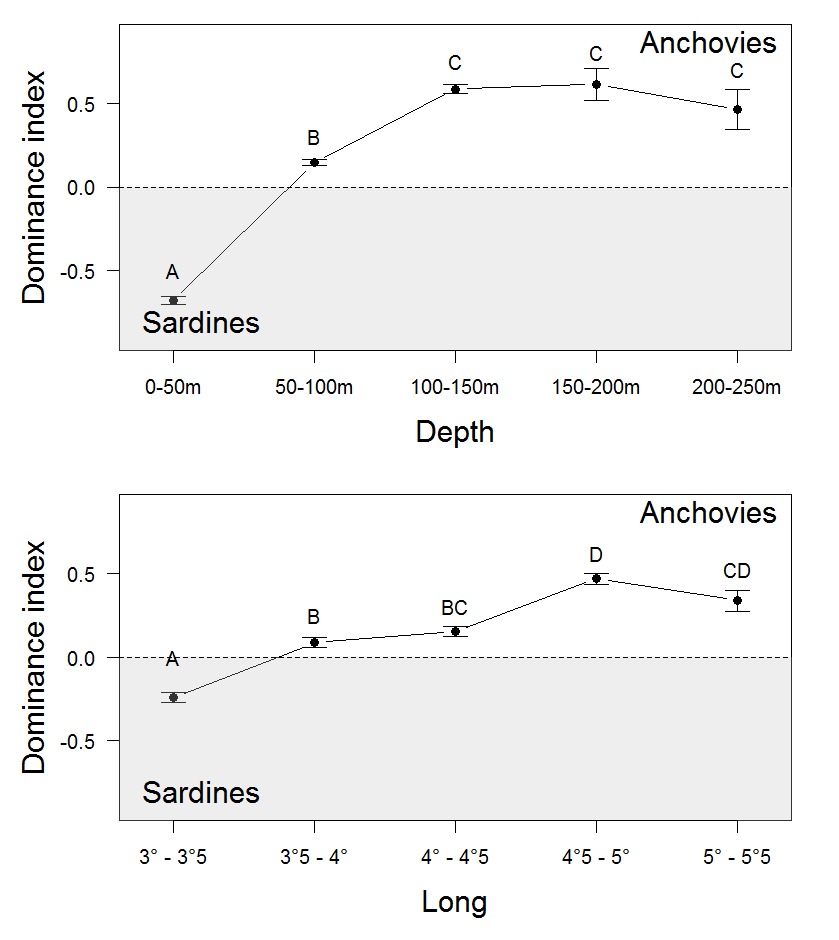
***

***Figure S8*** Dominance index depending on depth or longitude strata. Relative biomass between anchovies and sardines according to depth and longitude. Positive values of the dominance index correspond to a higher biomass of anchovies, while negative ones correspond to a higher biomass of sardines. Points sharing no common letters are significantly different. The 200-250m stratum is given for information, but it should be noted that the sampling effort in that zone was limited and that biomass densities are small.
